# Effect of conjugated linoleic acid on inhibition of prolyl hydroxylase 1 in hearts of mice

**DOI:** 10.1186/1476-511X-11-22

**Published:** 2012-02-07

**Authors:** Jize Zhang, Defa Li

**Affiliations:** 1 National Key Laboratory of Animal Nutrition, College Animal Science and Technology, China Agricultural University, Beijing 100193, People's Republic of China; 2 College Animal Science and Technology, 2-Yuan-Ming-Yuan West Rd., Haidian District, Beijing 100193, China

**Keywords:** CLA, HIF-2α, PDK4, PPARα

## Abstract

**Background:**

Results from different trails have provided evidence of protective effects of *cis-*9,*trans*-11-conjugated linoleic acid (CLA) on cardiovascular diseases. But the inhibition of prolyl hydroxylase 1 (PHD1) associated with induction of hypoxia inducible factors (HIFs) by CLA in these protective effects has never been reported before. The objective of this study was to evaluate if the two predominant *cis-*9,*trans*-11 (c9, t11), *trans*-10,*cis*-12 (t10, c12) CLA isomers and mixture of these two isomers can inhibit PHD1 with induction of HIFs in myocardium in mice and subsequent effects on myocardium metabolism.

**Results:**

CLA mixture and c9, t11 CLA inhibited PHD1 protein expression and increased the levels of protein and mRNA in HIF-2α in myocardium in mice. Meanwhile, CLA mixture and c9, t11 CLA also elevated the expression of HIF related transcriptional factors like PDK4 and PPARα. The reprogramming of basal metabolism in myocardium in mice was shown on increasing of GLUT4 gene expression by c9, t11 CLA supplemented group. UCP2 was increased by CLA mixture and c9, t11 CLA for attenuating production of ROS.

**Conclusion:**

CLA mixture and c9, t11 CLA could inhibit PHD1 and induce HIF-2α in myocardium in mice, which is associated with upregulation of PDK4 by activation of PPARα. This process also implies a reprogramming of basal metabolism and oxidative damage protection in myocardium in mice. All the effects shown in hearts of mice are due to c9, t11 CLA but not t10, c12 CLA.

## Background

Heart disease like myocardial infarction (MI) or acute myocardial infarction (AMI) and heart ischemia commonly are known as cardiovascular diseases (CVDs), which are the interruption of blood supply to part of the heart, causing heart cells to die. In 2008, an estimated 17.3 million people died from CVDs in the world, in which over 80% of CVD deaths take place in low-and middle-income countries [1].

Oxygen availability is insufficient when inadequate blood supply happens. Cells undergo adaptive changes in gene expression that promote survival in low oxygen (hypoxic) environment. Cellular adaptation to oxygen availability is mediated by the hypoxia inducible factors (HIFs), a member of the basic helix-loop-helix-PAS superfamily which transactivate a host of genes in the nucleus involved in the adaption of hypoxic stress [2]. HIF consists of an unstable α subunit and a stable β subunit that binds DNA at specific locations termed hypoxia response elements (HERs) to regulate many genes expression related to hypoxia [3]. HIF-α subunit is regulatory and unique to the hypoxic response. HIF-β subunit is constitutive and also involved in xenobiotic response. Three different genes encoding HIF-α subunit are found in mammals: HIF-1α, HIF-2α and HIF-3α [2]. HIF-α proteins are maintained at low steady-state level under normoxic condition via hydroxylation by HIF prolyl hydroxylases (PHDs) [4]. Among these three HIF-α isoforms, HIF-2α in particular shows a unique ability to induce metabolic reprogramming, which ultimately makes mitochondrion harmless but less active in certain conditions by regulating expression of numerous genes [5]. PHDs are 2-oxoglutarate dioxygenases, which are present in three forms in mammals, designated PHD1, PHD2 and PHD3 [6]. Hydroxylated HIF recruits the E3-ubiquitin ligase, von Hippel-Lindau protein (pVHL) [7,8], which in turn tags HIF with ubiquitin groups and targets it for degradation by proteasome [9,10].

Many cardiovascular diseases including anemia, myocardial infarction and stroke are linked to inadequate tissue oxygen. So, up-regulation of HIFs by inhibition of PHDs may have beneficial effect on therapy for hypoxia dependent process involved in cardiovascular disease [10]. The availability of less cumbersome non-toxic inhibitors of PHDs has been proved very useful for therapeutic intervention [11-13].

Conjugated linoleic acid (CLA) refers to a group of positional and geometric isomers of the essential fatty acid-linoleic acid (LA), which is produced by the bacterial biohydrogenation of linoleic acid in the gut of ruminant animals via an enzymatic isomerase reaction [14]. CLA is found naturally in food products from these animals predominantly as the *cis*-9,*trans*-11 form, whereas synthetic CLA preparations consist of a few different isomers with approximately equal amount of *cis*-9,*trans-*11 and *trans*-10,*cis*-12 CLA [15].

Since be found from 1980s, many research has been done with biological functions of these two predominant isomers of CLA. These isomers are both biologically active and known to have different physiological effects [16]. The original discovery of CLA was as an anticancer component, which was proven to be an effective prevention tool in a number of animal cancer models, such as skin, colon, mammary, lung and liver [17,18]. Both isomers have proven to be effective in prevention of cancer, while others reported differences in anti-cancer activities between these isomers [19,20]. t10, c12 CLA was reported to prevent cardiovascular disease [21,22] and reduce body fat in animals [23], which is one of the most important activities of CLA. While c9, t11 CLA was not responsible for these effects [24,25]. There was also research about CLA that it can reduce inflammatory and improve immune response [26-28]. But the controversy was that t10, c12 CLA induced inflammatory responses in white adipose tissue [29]. Besides positive effects induced by CLA, there were also negative effects reported for CLA, especially t10, c12 CLA. Mice fed with CLA mixture or t10, c12 CLA resulted in lipodystrophy, hyperinsulinemia and liver steatosis, whereas, c9, t11 CLA had no negative effect like t10,12 CLA [30-32].

There was research reported that c9, t11 CLA in adipose tissue was associated with a lower risk of MI in basic [33]. But the mechanism of this protective effect is still not fully defined. We estimated that CLA may have influence on PHDs, which is related to the protective effect on myocardium. The mice model was used by supplementing with individual isomers of CLA and the mixture of these two isomers in the diet to determine if these effects are associated with role of prolyl hydroxylase inhibitor on myocardium.

## Material and method

### Material

Semipurified diet (TD.04460) was from Harlan Teklad (Madison, WI). Antibodies for prolyl hydroxylase 1 (PHD1) and glyceraldehyde 3-phosphate dehydrogenase (GAPDH) were purchased from Bethyl (Montgomery, TX). Antibodies for hypoxia inducible factor-2 alpha (HIF-2α), peroxisome proliferator-activated receptor alpha (PPARα) and secondary antibody coupled to horse radish peroxidase (HRP) were purchased from Abcam (Cambridge, MA). Other solvents used were purchased from either Sigma Chemical Co. (St. Louis, MO) or Fisher Scientific (Pittsburg, PA).

### Animal and diet

Forty six-month old female ICR mice were randomized into four groups of ten mice each. Animals were housed in individual wire-bottomed cages in a temperature-controlled, humidified room maintained on a 12 h on/off light cycle and were provided free access to food and water. The mice were fed a semipurified powered diet (TD.04460) (Table 1). Diets were prepared at the beginning of the study and kept at -20°C until use. Fresh diet was provided three times a week. After 1 week adaptation, animals were fed one of the following diets; control, 0.5% CLA mixture (Mixture), and 0.20% *cis*-9,*trans*-11 CLA (c9, t11 CLA), or 0.20% *trans*-10,*cis*-12 CLA (t10, c12 CLA). 0.20% of the individual isomers are equivalent to the levels found in 0.5% CLA mixture diet. Body weight was measured weekly. All CLA preparations were purchased from Natural ASA (Hovdebygda, Norway). The composition of the CLA preparations is given in Table 2.

**Table 1:** TD.04460 Basal Mix (94%)

Formula	g/Kg
Casein, "Vitamin-Free" Test	206.39
L-Cystine	3.192
Corn Starch	357.377
Maltodextrin	140.43
Sucrose	93.1
Soybean Oil	95.75
Cellulose	53.2
Mineral Mix, w/o Ca (04374)	37.24
Vitamin Mix, AIN-93-VX (94047)	10.64
Choline Bitartrate	2.66
TBHQ, antioxidant	0.021

**Table 2:** Composition (%) of CLA Preparations Used in this study^a^

	CLA preparation		
**FA**	**> 90% c9, t11**	**> 90% t10, c12**	**Mixture**

c9, t11 CLA	88.6	5.7	44.3
t10, c12 CLA	3.0	89.5	43.9
Oleic acid (18:1)	3.7	1.6	5.5
other	3.7	3.2	6.3

^a^Analytical data provided by Natural ASA (Hovdebygda, Norway)

### Sacrifice and tissue sample collection

After 8 weeks, animals were sacrificed by CO_2 _asphyxiation after 4 h fasting. Hearts were removed and frozen by immersion in liquid N_2_, stored at -80°C until needed for analysis.

### mRNA expression analysis

From frozen hearts, mRNA expression levels of hypoxia inducible factor-1 alpha (HIF-1α), endothelial PAS domain-containing protein 1 (EPAS1), aryl hydrocarbon receptor nuclear translocator 2 (ARNT2), egl nine homolog 2 (EGLN2), pyruvate dehydrogenase kinase 4 (PDK4), peroxisome proliferator-activated receptor alpha (PPARα), carnitine palmitoyltransferase 1b (CPT1b), glucose transporter type 4 (GLUT4), uncoupling protein 2 (UCP2) were analyzed by real-time PCR using StepOnePlus™ Real-Time PCR System with StepOne Software v2.0 (Applied Biosystems, Foster City, CA), TaqMan^® ^Gene Expression Master Mix (Applied Biosystems, Foster City, CA) and TaqMan Gene Expression Assays (Applied Biosystems, Foster City, CA). Total RNA from heart was isolated with Trizol Reagent (Invitrogen, Carlsbad, CA) according to the manufacturer's instructions. Total RNA was reverse-transcribed using High Capacity cDNA Reverse Transcription kit (Applied Biosystems, Foster City, CA). The reference sequence for the mRNAs, the housekeeping gene control (GAPDH) is shown in Table 3.

**Table 3:** Information of gene primers used for RT-PCR

Gene	Assay ID	RefSeq	Exon Boundary	Assay Location
ARNT2	Mm00476004_m1		12-13	1343
CPT1b	Mm00487200_m1	NM_009948.2	17-18	2228
EGLN2	Mm00519067_m1	NM_053208.4	2-3	1227
EPAS1	Mm01236112_m1	NM_010137.3	6-7	1192
GLUT4	Mm00436615_m1	NM_009204.2	9-10	1328
HIF-1α	Mm00468869_m1	NM_010431.2	4-5	879
HIF-3α	Mm00469373_m1		10-11	1407
PDK4	Mm01166879_m1	NM_013743.2	3-4	454
PPARα	Mm00440939_m1	NM_011144.6	7-8	1371
UCP2	Mm00627597_m1	NM_011671.4	1-2	119
GAPDH		NM_008084.2		

### Western blot analysis

Tissue lysates were prepared in homogenization buffer [50 mM Tris-HCl pH 7.5, 10%(v/v) glycerol, 5 mM magnesium acetate, 0.2 mM EDTA, 0.5 mM dithiothreitol (DTT), 1 mM phenylmethylsulfonyl fluoride (PMSF)] and centrifuged at 12,000 r.p.m. for 20 min at 4°C. Protein concentration was determined by Bio-Rad protein assay (Hercules, CA) using 1.4 mg/mL bovine serum albumin (BSA) as the standard. Proteins (25 μg) from each sample were separated on SDS-PAGE and transferred to PVDF membranes (Millipore, Billerica, MA). After blocking with a specific primary antiserum in Tris buffered saline (TBS) containing 0.05% Tween-20 (TBS-T) and 5% non-fat dry milk at 4°C overnight, the membrane was incubated with each antibody (anti-PHD1, anti-HIF-2α, anti-PPARα, anti-GAPDH) at 4°C overnight. Finally, after three washes with TBS-T, the blots were incubated with secondary antibody coupled to HRP for 1 h at room temperature, visualized using ECL Plus Western Blotting Detection System (GE Healthcare, Buckinghamshire, UK) and quantified by Kodak Image Station Software (Scion, Frederick, MD). Relative protein levels were determined by comparison to GAPDH band intensity, which was used as internal control for western blotting analysis.

### Statistical analysis

Data are shown as means and standard errors. All analyses were carried out using SAS software (Version 9.2, SAS institute Inc., Cary, NC) by one-way analysis of variance (ANOVA) followed by Tukey's multiple comparison test.

## Results

### Body weight

No significant difference in body weight was observed with the exception of week 4, where CLA mixture-fed animal had significant lower weight compared to control (Table 4).

**Table 4:** Effect of individual isomers of CLA and CLA mixture on body weight in the mice

Diet	BW0	BW1	BW2	BW3	BW4	BW5	BW6	BW7	BW8
Control	25.90 ± 0.65^a^	29.68 ± 0.44^a^	29.49 ± 0.51^a^	30.19 ± 0.60^a^	30.40 ± 0.64^a^	30.29 ± 0.41^a^	31.94 ± 0.44^a^	32.93 ± 0.52^ab^	33.17 ± 0.58^ab^
CLA mixture	25.90 ± 0.65^a^	28.58 ± 0.46^a^	28.03 ± 0.58^a^	28.68 ± 0.68^a^	28.44 ± 0.77^b^	29.29 ± 0.77^a^	32.00 ± 0.73^a^	32.30 ± 0.71^b^	31.88 ± 0.79^b^
c9, t11 CLA	25.92 ± 0.65^a^	29.82 ± 0.61^a^	29.41 ± 0.68^a^	30.23 ± 0.62^a^	29.93 ± 0.67^ab^	30.04 ± 0.82^a^	32.36 ± 0.69^a^	34.36 ± 0.82^a^	34.29 ± 0.89^a^
t10,12 CLA	25.63 ± 0.63^a^	29.31 ± 0.41^a^	28.92 ± 0.43^a^	29.79 ± 0.45^a^	29.20 ± 0.32^ab^	30.93 ± 0.32^a^	32.82 ± 0.41^a^	34.05 ± 0.44^ab^	33.26 ± 0.60^ab^

Body weight (BW) in grams was measured weekly (0-8 weeks). Female mice were fed one of the treatment diets for 8 weeks: control, 0.5% CLA mixture, 0.2% c9, t11 CLA and 0.2% t10, c12 CLA. Values within a column not sharing a common superscript letter indicate significant difference at *P *< 0.05. Numbers are mean ± S.E. (n = 10)

### Gene expressions of HIFs and PHD1

**Figure 1:**
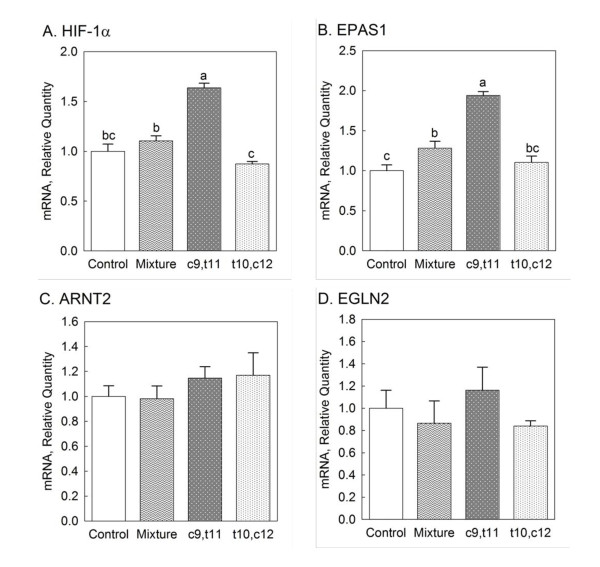
**Effects of individual isomers of CLA and CLA mixture on gene expressions of HIFs and PHD1 from the heart: (A) HIF-1α, (B) EPAS1 (HIF-2α), (C) ARNT2 (HIF-β) and (D) EGLN2 (PHD1)**. Female mice were fed one of the treatment diets for 8 weeks: control, 0.5% CLA mixture, 0.2% c9, t11 CLA and 0.2% t10, c12 CLA. Different letters in each figure indicate significant difference at *P *< 0.05. Numbers are mean ± S.E. (n = 5).

The effect of individual isomers of CLA and CLA mixture on gene expressions of three types of HIF-α subunits, HIF-β subunit and HIF-α subunit hydroxylase were determined. As shown in Figure 1A, c9, t11 CLA had effect on HIF-1α gene expression. As seen in Figure 1B, 1-fold increase in EPAS1 (HIF-2α) gene expression was noted in mice fed with c9, t11 CLA and the same effect was also seen in CLA mixture-fed mice (*P *< 0.05). HIF-3α gene expression was undetermined among all treatments. ARNT2 (HIF-β) and EGLN2 (PHD1) gene expressions were unaltered by all treatment (Figure 1C and 1D).

### Protein expressions of HIF-2α and PHD1

**Figure 2:**
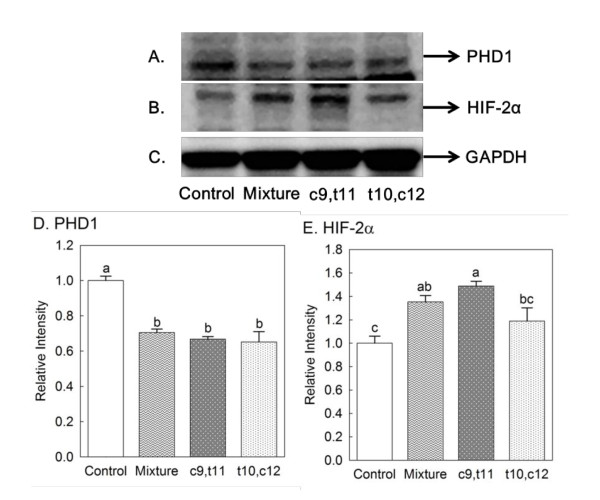
**Effects of individual isomers of CLA and CLA mixture on protein expressions from the heart: (A) PHD1 and (B) HIF-2α; relative band intensity statistical results: (D) PHD1 and (E) HIF-2α**. (C) GAPDH was used as internal control for western blotting analysis. Female mice were fed one of the treatment diets for 8 weeks: control, 0.5% CLA mixture, 0.2% c9, t11 CLA and 0.2% t10, c12 CLA. Different letters in each figure indicate significant difference at *P *< 0.05. Numbers are mean ± S.E. (n = 3).

After determining the effect of individual isomers of CLA and CLA mixture on HIFs and PHD1 in mRNA level, protein expressions of HIF-2α and PHD1 from animals were also determined. As shown in Figure 2A and 2C, the highest PHD1 protein expression was observed in control-fed animal compared to other groups. The effect of CLA mixture and c9, t11 CLA on increasing HIF-2α protein expression was shown in Figure 2B and 2E.

### Gene and protein expressions of PPARα pathway

**Figure 3:**
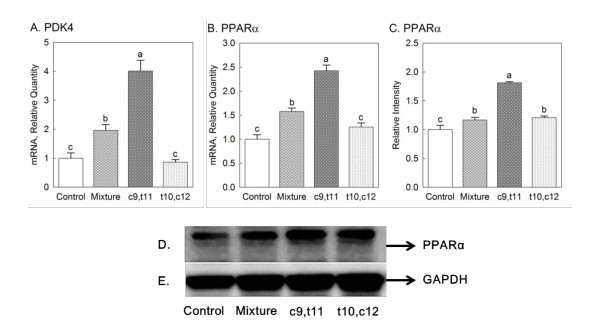
**Effects of individual isomers of CLA and CLA mixture on gene and protein expressions of PPARα pathway from the heart: (A) PDK4 gene expression, (B) PPARα gene expression, (C) protein relative band intensity statistical result of PPARα and (D) PPARα protein expression**. (E) GAPDH was used as internal control for western blotting analysis. Female mice were fed one of the treatment diets for 8 weeks: control, 0.5% CLA mixture, 0.2% c9, t11 CLA and 0.2% t10, c12 CLA. Different letters in each figure indicate significant difference at *P *< 0.05. Numbers are mean ± S.E. (n = 5 for gene expressions and n = 3 for protein expression).

As recently noted [5], enhanced expressions of PDK4 and PPARα associated with stabilized HIF-2α were shown in mouse skeletal muscle. To determine if the effect of individual isomers of CLA and CLA mixture on stabilization of HIF-2α might be mediated by PPARα pathway, PDK4 and PPARα expressions in myocardium in mice were measured. Compared with control group (*P *< 0.05), a significant elevation of PDK4 gene expression was evident in mice fed with CLA mixture and c9, t11 CLA, as shown in Figure 3A. The same trend of PPARα gene expression was also observed as PDK4 gene expression (*P *< 0.05) (Figure 3B). Similar as PPARα gene expression, CLA mixture, c9, t11 CLA but also 10,12 CLA increased PPARα protein expression in comparison with control (Figure 3C and 3D).

### Gene expressions of glucose and lipid metabolism enzymes

**Figure 4:**
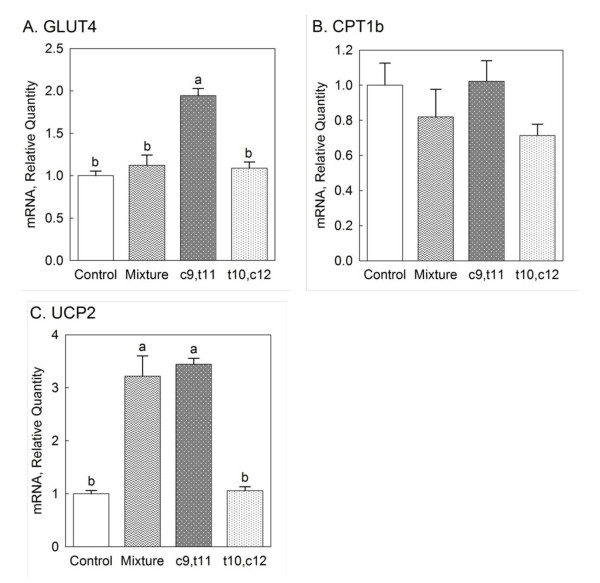
**Effects of individual isomers of CLA and CLA mixture on gene expressions of glucose and lipid metabolism enzymes from the heart: (A) GLUT4, (B) CPT1b and (C) UCP2**. Female mice were fed one of the treatment diets for 8 weeks: control, 0.5% CLA mixture, 0.2% c9, t11 CLA and 0.2% t10, c12 CLA. Different letters in each figure indicate significant difference at *P *< 0.05. Numbers are mean ± S.E. (n = 5).

As a marker for glucose metabolism, GLUT4 gene expression was only increased by c9, t11 CLA fed group compared with the other groups (*P *< 0.05) (Figure 4A). For a key enzyme of fatty acid β-oxidation, CPT1b gene expression was not altered by all treatments (Figure 4B). CLA mixture and c9, t11 CLA significantly increased UCP2 gene expression (*P *< 0.05) relative to control and t10, c12 CLA fed animals (Figure 4C).

## Discussion

In this study, CLA mixture and the two predominant isomers of CLA showed the ability to inhibit PHD1 protein expression. But interestingly, only CLA mixture and c9, t11 CLA played a role in stabilizing HIF-2α in the normoxia environment and affected expressions of HIF related transcriptional genes with inhibition of PHD1 in hearts of mice. This is a new discovery of well-studied CLA on myocardium protective effect.

One of the important effects of CLA is on decreasing body weight gain, which was proved in various animal models like mice and rats [25,34]. However, these findings were shown when those animals were given high amount of CLA, in which t10, c12 CLA was contributed to this effect [35,36]. When animals were supplemented with low amount of CLA mixture (≤ 0.5% in the diet) or the two predominant isomers of CLA, the body weights were not affected [37-39], which is consistent with our study.

Since PHD1 is a direct oxygen sensor, alternation of expression of PHD-1 is expected to change hypoxic adaptation [40]. Most of the PHD inhibitors reported so far, e.g. DMOG [41], 3,4-DHB [42], FG-0041 [43] are chemicals. None of them is from natural food like CLA which is contained in milk or beef. Inhibition of PHD1 associated with stabilized expression HIF-2α expression in normoxia condition was seen in CLA mixture and c9, t11 CLA fed mice, which is consistent with a former study that mice lacking PHD1 (PHD1-null mice) showed significant expression of HIF-2α in the absence of hypoxia [5].

Specifically, CLA mixture and c9, t11 CLA increased HIF-α expression like HIF-1α and HIF-2α in mRNA or protein level. The protective effect of HIFs within different tissues has already been noted before [5,44,45]. However, there is a debate about the expression of HIF-α in mRNA and in protein level. Research indicated that there was no significant increase in the mRNA expression of EPAS1 [46,47]. This may be due to posttranscriptional regulation as found in specific cell types [48,49]. From the results showing in the experiment, the regulation of expression of EGLN2 (PHD1) in different levels may share the same mechanism as HIF-α. Interesting, t10, c12 CLA also down-regulated PHD1 protein expression, but it was not as potent as 19, t11 CLA on EPAS1 expression, which may be the difference of bioactivity of these two isomers of CLA. In general, HIF-β is constitutively expressed and heterodimerizes with HIF-α subunit in the nucleus to form a complex, which binds to hypoxia-responsive elements in enhancers and promoters of oxygen-responsive genes under hypoxic conditions [47]. Consistent with the former research, the expression of ARNT2 was not altered by all treatments in this study.

The possible mechanism inducing protective effect of HIFs, especially HIF-2α, is linked to an increase in the expression of PDK4 [50]. In the normoxic environment, pyruvate enters the tricarboxylic acid (TCA) cycle inside the mitochondrion where it generates ATP in the presence of oxygen. But with the inhibition of PHD1 or in the hypoxia environment like heart ischemia, entry of pyruvate is restricted by expression of PDK4, which is associated with induction of HIF-2α [5,51]. In this study, PDK4 gene expression was upregulated in mice fed with CLA mixture and c9, t11 CLA.

PPARα is known to activate the PDK4 gene [51]. Research on the hibernating mammal model also showed the level of PDK4 mRNA increased greatly by activation of PPARα during hibernation [52]. Hearts from PPAR agonist clofibrate-treated rats had an improved recovery of post-ischemic contractile function and reduced ischemia/reperfusion (I/R)-induced infarct size. The coincident upregulation of PPARα and PDK4 in PHD1-deficient was also demonstrated by knockdown of PPARα in muscles of PHD1-deficient mice *in vivo *and feeding wild-type mice with PPARα agonist [5]. In this study, we found that CLA mixture and the two predominant isomers of CLA significantly increased PPARα protein expression. This result, combined with PDK4 gene expression and HIF-2α expression, suggests that PPARα activated by CLA mixture and c9, t11 CLA, with induction of HIF-2α, can initiate PDK4 gene expression.

Induction of HIF-2α by inhibition of PHD1 implies a reprogramming of basal metabolism in mice, especially in energy production and utilization like glucose metabolism and fatty acid metabolism. Glycolytic flux was increased in mouse muscle fiber in low oxygen conditions [5]. As a marker for glucose metabolism, c9, t11 CLA fed mice increased GLUT4 gene expression significantly in this study. Combined, c9, t11 CLA may induce more glucose entering cell cytoplasm to generate ATP by glycolysis when PHD1 was inhibited. But for t10, c12 CLA, which promoted dysregulation of lipid and glucose metabolism in hepatic tissue while c9, t11 CLA had not effect [32]. There was also another research reported that mice supplemented 0.5% CLA mixture showed different effect on GLUT4 gene expression in adipose and skeletal muscle tissue [53]. All the evidences showed in experiments suggested that the two predominant isomers of CLA may have diverse effect on glucose metabolism in different tissues. CPT1b is known as a rate-limiting enzyme in fatty acid β-oxidation which transfers fatty acids through mitochondrial membrane to be oxidized within the mitochondrial matrix [54]. A former research showed that fatty acid oxidation was not altered in PHD1-deficient mice [5], which is consistent with our research that CPT1 mRNA expression was not changed during PHD1 inhibition by CLA mixture and the two predominant isomers of CLA supplementation in the diet.

As an important energy expenditure parameter, the effect of uncoupling proteins (UCPs) has become prominent in the field of thermogenesis, especially UCP1 [55]. But for UCP2 and UCP3, there is a consensus that the primary function of UCP2 and UCP3 is to attenuate mitochondrial production of free radical to protect against oxidative damage, degenerative disease and aging rather than to promote gross thermogenesis or energetic inefficiency [56,57]. Oxidative damage caused by reactive oxygen species (ROS) is produced in mitochondrion, which can trigger the toxic effects living with oxygen. In contrast, oxygen depletion (hypoxia) also increases mitochondrial ROS that is detrimental to cells unless attenuated [58]. In this study, the UCP2 gene expression in mice fed with CLA mixture and c9, t11 CLA was elevated greatly, which is consistent with former research done on effect of elucidating UCP2 on attenuating ROS production [59,60].

## Conclusion

To our knowledge, this study is the first to examine the effect of CLA on inhibition of PHD1 *in vivo*. One of the two predominant isomers of CLA- c9, t11 CLA showed more potent effect than the other CLA isomer- t10, c12 CLA. This inhibitory effect is associated with induction of HIF-2α. Several lines of evidence suggest the protective effect of c9, t11 CLA on inhibition of PHD1 plays a part by upregulation of PDK4 gene expression, which is activated by PPARα. This process can imply a reprogramming of basal metabolism in hearts of mice by increasing glycolysis. Meanwhile, c9, t11 CLA also increased UCP2 gene expression to attenuate the damage of ROS. This study provides a new interpretation of protective effect in myocardium *in vivo *by CLA, especially one of the predominant isomers-c9, t11 CLA.

## Competing interests

The authors declare that they have no competing interests.

## Authors' contributions

JZZ and DFL conceived the study and design. JZZ has performed all experiments and analyzed data, interpreted results and written the final draft of this manuscript. All authors have read and approved the final manuscript.
